# Pneumonia

**Published:** 1886-01

**Authors:** 


### PNEUMONIA.

The prevalence of pneumonia, its rapid increase and fatal consequences in many instances, have led a number of our abler physicians, to carefully investigate the peculiaralities of this alarming disease, and some of them have published the result of their observations in a way to benefit the public, not only by pointing out the best methods of prevention, but likewise of treatment, in the event of its occurence.

Dr. John T. Nage, Deputy Register of Records of the Health Board of the City of New York, has given much attention to the disease and has prepared valuable statistical tables concerning it.

"The prevalence of pneumonia," he says, "may be owning to a lack of ozone in the air, or it may be because there is too much ozone. Sudden changes of weather and high winds, particularly from the north and east, certainly have much to do with it and draughts of all kinds are bad and should be avoided. Smoking may be a predisposing cause, as tobacco is certainly an irritant. Anything which irritates the lungs should be avoided. If people would breathe through the nose instead of through the mouth, especially when in the open air or facing a cold wind, the lungs would be less irritated.

"One great cause of the fearful death-rate among children from this disease is undoubtedly, the criminally foolish way in which they are dressed. Many mothers seem more anxious to make their children look pretty than to dress them comfortably. On a par with this is the folly of low-necked dresses among women as viewed from a health standpoint. Ladies so dressed will rush from a heated ballroom or theatre into the open air and then wonder that they have colds or pneumonia. Wear seasonable underclothing and don't remove your heavy flannels too early in the spring or defer putting them on until too late in the fall. I should not advise people to coddle themselves, but one should dress according to the seaso[illegible] and should cover the body evenly. Add to this a proper regard for the general health and an avoidance of draughts and one need not worry much about pneumonia."

Prof. A. L. Loomis, in his "Practice of Medicine," says: "It is a well-known fact that the disease attacks the poor oftener than the rich, the private oftener than the officer, the sailor on shore oftener than on ship, the soldier oftener than the civilian at the same post. It is unknown in the polar regions and common on the Mediterranean, increasing in a direct ratio from the poles to the equator. Elevation above the sea predisposes to it; north and east winds favor its development; rainy seasons or damp and marshy districts do not seem to influence it. Periods of steady and extreme cold have little effect except upon the old, but sudden changes are very disastrous. The first predisposing cause is age, the disease being most common in early childhood, from twenty to forty and after sixty. The proportion of male to female victims is as three to one. Any general condition of the body which debilitates is a predisposing cause. The complications which render the disease so dangerous are those which diminish the nerve supply or weaken the muscular power of the heart. Bad sewerage and miasmatic influence are potent causes of the disease."

Pneumonia usually begins with a chill, intense and prolonged, generally at night, and followed by a correspondingly high fever and sharp pains in the sides. The disease is very rapid in its progress, reaching a crisis in from five to six days and sometimes causing death within three days. Usually but one lung is affected and often the disease is confined to a single lobe. A person may have "double pneumonia," or pneumonia of both lungs, and recover from it, but the chances are against him. When the disease spreads to all of the lung lobes death is certain, as the patient cannot breathe and dies of suffocation. The diseased lung, at first inflamed, soon becomes hard and leathery and incapable of performing its natural functions. A curious fact is that usually no second chill occurs when another lobe is attacked, and there appears to be no relation between the amount of lung affected and the intensity of the symptoms. All physicians agree in saying that the disease is not contagious, but may be epidemic, and it has been noticed that it is developed under the same conditions as diphtheria—that is, the conditions which produce diphtheria in the young are apt to cause pneumonia among adults.

Dr. J. R. Leaming, special consulting physician in chest diseases in St. Luke's Hospital, has published a little pamphlet concerning it, entitled, "Endemic Pleuro-Pneumonia, as seen in New York during the past ten or twelve years." In that pamphlet Dr. Leaming holds to the theory that the pneumonia of the present day, or pleuro-pneumonia, as he calls it, is the same as the epidemic which caused such havoc among the troops in Canada during the war of 1812-15.

That the weather has much to do with pneumonia is apparent. The number of deaths in New York City for the first seven months of the present year were as follows: January, 375; February, 486; March, 587; April, 512; May, 337; June, 229; July, 150. After August there is usually a steady increase until March, the most fatal month of the year. The death rate, too, is very high. The statistics so far published, both in hospitals and private practice, show an average death rate of at least 20 per cent., or one in five of those attacked.

The theories concerning the nature of the disease itself are many and varied. Some physicians hold that pneumonia is only a local manifestation of a general disease, others that it is a specific disease caused by a specific poison, while still others hold as tenaciously to the germ theory.

Without speculating upon these different theories, from what has been said in which all agree, it is plain that anything which lowers the vitality of the system, is conducive to the disease and should be carefully avoided. Over work, either physical or mental, has much to do with it and this explains why so many business men and brain-workers become its victims. Sudden changes of the weather and draughts of all kinds are also to be guarded against. In a word, live temperately, dress warmly, avoiding all manner of imprudencies, and you need have no fear of pneumonia.

